# Injectable selenium-containing polymeric hydrogel formulation for effective treatment of myocardial infarction

**DOI:** 10.3389/fbioe.2022.912562

**Published:** 2022-07-11

**Authors:** Cui Yang, Chunyan Zhu, Yanling Li, Zibiao Li, Zhenghao Zhang, Jiajia Xu, Minwei Chen, Runjing Li, Shixiao Liu, Yunlong Wu, Zhengrong Huang, Caisheng Wu

**Affiliations:** ^1^ Xiamen Key Laboratory of Cardiac Electrophysiology, Department of Cardiology, School of Medicine, Xiamen Institute of Cardiovascular Diseases, The First Affiliated Hospital of Xiamen University, Xiamen University, Xiamen, China; ^2^ Fujian Provincial Key Laboratory of Innovative Drug Target Research and State Key Laboratory of Cellular Stress Biology, School of Pharmaceutical Sciences, Xiamen University, Xiamen, China; ^3^ Institute of Materials Research and Engineering (IMRE), Agency for Science, Technology, and Research (A*STAR), Singapore, Singapore; ^4^ Institute of Sustainability for Chemicals, Energy and Environment (ISCE2), Agency for Science, Technology, and Research (A*STAR), Singapore, Singapore

**Keywords:** poly[di-(1-hydroxylyndecyl) selenide/polypropylene glycol/polyethylene glycol urethane], myocardial infarction, thermosensitive injectable hydrogel, selenium-containing polymers, inflammation, fibrosis

## Abstract

Myocardial infarction (MI) is a serious threat to people’s life and health, which is significantly hindered by effective treatment formulations. Interestingly, our recent endeavour of designing selenium-containing polymeric hydrogel has been experimentally proved to be helpful in combating inflammatory responses and treating MI. The design was inspired by selenium with anti-inflammatory and anti-fibrosis activities, and the formulation could also serve as a support of myocardial tissue upon the failure of this function. In details, an injectable selenium-containing polymeric hydrogel, namely, poly[di-(1-hydroxylyndecyl) selenide/polypropylene glycol/polyethylene glycol urethane] [poly(DH-SE/PEG/PPG urethane)], was synthesised by combining a thermosensitive PPG block, DH-Se (which has oxidation-reduction properties), and hydrophilic PEG segments. Based on the established mouse model of MI, this formulation was experimentally validated to effectively promote the recovery of cardiac function. At the same time, we confirmed by enzyme-linked immunosorbent assay, Masson staining and Western blotting that this formulation could inhibit inflammation and fibrosis, so as to significantly improve left ventricular remodelling. In summary, a selenium-containing polymeric hydrogel formulation analysed in the current study could be a promising therapeutic formulation, which can provide new strategies towards the effective treatment of myocardial infarction or even other inflammatory diseases.

## 1 Introduction

Myocardial infarction (MI) is one of the most common cardiovascular diseases (Zhao et al., 2019). Considering its pathogenesis, MI is generally attributed to the rapid reduction or interruption of coronary artery blood supply, resulting in severe and persistent acute ischemia of the corresponding myocardium, leading to the necrosis of myocardial cells (Thygesen et al., 2007). Currently, MI and subsequent heart failure are still the leading causes of death in many countries, with high associated morbidity and mortality rates, which is a serious public health issue that needs to be addressed (Reed et al., 2017). However, owing to the presence of negative ventricular remodelling, there is still a progressive decline in the cardiac function of MI survivors, accompanied by irreversible myocardial necrosis and cardiac function decline (Lloyd-Jones et al., 2010). Therefore, it is particularly important to take timely and effective measures to control the condition of patients with myocardial infarction. Among them, drug therapy is the basic treatment method for clinical treatment of myocardial infarction, which can alleviate the symptoms of patients to a certain extent, but there are still few effective therapeutic drugs at present.

In recent decades, targeted therapy on promoting myocardial repair and regeneration after MI has gained much prominence. Thus far, the therapeutic methods that have been explored to improve myocardial function include direct injection of bioactive substances and the use of biomaterials (Amado et al., 2005; Garbern and Lee, 2013; Jackman et al., 2015; Korf-Klingebiel et al., 2015; Tang, 2015; Rebouças et al., 2016; Singh et al., 2016; Yi et al., 2019). Direct injection of bioactive substances has many drawbacks, such as short half-life of biomolecules *in vivo*, non-specific delivery, and poor localisation of target regions (Peña et al., 2018). The use of biomaterials can effectively overcome these limitations, thereby protecting the delivered materials from degradation, improving targeted delivery, and increasing cell viability (Jackman et al., 2015; Truskey, 2016). Currently, biomaterials used in cardiac tissue engineering mainly include injectable hydrogels, patches, and cell sheets (Tous et al., 2011; Castells-Sala and Semino, 2012; Ye et al., 2013; Matsuura et al., 2014). Among them, patches and cell sheets are difficult to translate into clinical applications because of the need for more invasive surgical interventions and complex processes (Peña et al., 2018). Injectable hydrogels can be injected into the myocardium through minimally invasive methods (such as catheter delivery) and are particularly suitable for cardiac regeneration and repair (Yoshizumi et al., 2016). Additionally, existing studies have proved that injectable hydrogels produce good results, with respect to the treatment of MI and the recovery of myocardial function (Matsumura et al., 2019). They can provide damaged cardiomyocytes a microenvironment similar to the extracellular matrix (ECM), in addition to mechanical support for weakened ventricular walls (Sacks et al., 2018). Moreover, they can be used as carriers for cells, proteins, drugs or other cell-growth promoting factors, to boost the repair of the injured myocardium after infarction (O'Neill et al., 2016).

The commonly used hydrogels include natural, synthetic, and mixed hydrogels. Although the development of multiple natural hydrogels, including collagen and chitosan, has led to much progress in cardiac engineering (McLaughlin et al., 2019; Liu et al., 2020), natural hydrogels have many shortcomings such as variable results and immunogenic risks, resulting in their inapplicability in clinical practice (Saludas et al., 2017). Therefore, the focus of efforts in cardiac tissue engineering has shifted to the use of synthetic materials, to meet the needs of specific applications by varying mechanical strength, porosity, degradation rate, and gelation rate (Peña et al., 2018). The risk of immune rejection after implantation of synthetic materials is lower, with only small differences between batches, owing to which injectable synthetic hydrogels are considered to have higher development value (Peña et al., 2018).

Inflammatory reaction is the initial stage of MI. Appropriate inflammatory reaction is conducive to the repair of the injured myocardium; however, continuous inflammatory reaction not only interferes with tissue repair but also induces cardiomyocyte apoptosis, necrosis, and fibrosis, ultimately leading to ventricular remodelling and cardiac insufficiency (Anzai, 2013; Frangogiannis, 2014). Therefore, studies have explored the use of anti-inflammatory drugs, anti-inflammatory hydrogels, or hydrogels combined with anti-inflammatory drugs and cells, to inhibit excessive early inflammation, for accelerating myocardial repair after MI (Shen et al., 2004; Ferrini et al., 2019; Chen et al., 2020). For instance, Ding et al. (2020) formulated an injectable hydrogel with the dual function of scavenging reactive oxygen species and generating oxygen using a hyperbranched polymer (HBPAK) aqueous solution containing thioketones (with antioxidant effect) and methacrylate hyaluronic acid, for MI treatment *in vivo*. Chen et al. (2020) used injectable thermosensitive hydrogels carrying colchicine, for repair after MI, while Chow et al. (2017) used PEG hydrogel to deliver erythropoietin (EPO) and cardiomyocytes derived from human induced pluripotent stem cells (hiPSC-CMs) to the infarcted myocardium, thereby promoting myocardial repair. Compared with hydrogels combined with anti-inflammatory drugs and cells, hydrogels without anti-inflammatory drugs or cells can be directly used for anti-inflammatory treatment, without considering the release and dosage of drugs or cells. Therefore, exploring more anti-inflammatory hydrogels without anti-inflammatory drugs or cells can provide more options for MI treatment.

This study aimed to establish a new thermosensitive hydrogel system that could be used as a potential biomaterial for myocardial infarction by introducing selenium-containing polymers. Selenium is one of the trace elements essential for the human body (Wang et al., 2017). As evidenced by prior studies, selenium plays a role in multiple biological activities such as antioxidation (Kiełczykowska et al., 2018), anti-inflammation (Dallak, 2017), and anti-fibrosis (Alehagen et al., 2018). Moreover, selenium-containing polymers exhibit low bond-energy, owing to the low electronegativity and atomic radius of selenium, which can react even under a mild stimulation (Xu et al., 2013). In view of these properties, selenium-containing polymers have become a potential medical biomaterial (Xu et al., 2013). Selenium-containing polymers can accomplish antioxidant and anti-inflammatory activities *in vivo*. Notably, with oxidative stress and inflammation playing an important role in the development and pathogenesis of MI (Hori and Nishida, 2009; Ding et al., 2020), it is worthwhile to explore the effect of selenium-containing polymers in treatment of MI. Accordingly, in our study, thermosensitive polyethylene glycol (PPG) fragments, di-(1-hydroxylyndecyl) selenide (DH-Se) fragments with oxidation-reduction properties, and hydrophilic polypropylene glycol (PEG) fragments were used to synthesise a novel thermosensitive selenium-containing polymer hydrogel, namely poly(DH-SE/PEG/PPG urethane) (Se-PEG-PPG), and the corresponding therapeutic effect post MI was verified *via* a mouse model. It was observed that Se-PEG-PPG exhibited good cardioprotective effects. It could promote the recovery of cardiac function, inhibit inflammatory reaction by reducing the expression of IL-6 and fibrosis by downregulating the expressions of fibrosis associated protein *in vivo*, and improve left ventricular remodelling. The current study investigated, for the first time, the effect of a novel selenium-containing polymer hydrogel in the treatment of MI. The synthesised Se-PEG-PPG was confirmed as a biomaterial with therapeutic effect on myocardial infarction, on the basis of its notable myocardial protective effects, as determined during the course of our study. It is expected that the results of our study will provide a reference for the selection of novel biomaterial and offer future research directions for the treatment of MI and other ischemic diseases.

## 2 Materials and methods

### 2.1 Materials

DH-Se was from Singapore A*STAR Materials Research and Engineering Institute; PEG, PPG, 1.6-hexadi-diisocyanate (HMDI), MeOD, and MTT [3-(4, 5-dimethyl-2-thiazole)-2, 5-diphenyl tetraazolium thiazole blue] were obtained from Sigma-Aldrich (United States). THF was purchased from Aladdin (China). FITC fluorescein isothiocyanate was obtained from Yeasen Biotech Co., Ltd. (China).

### 2.2 Animals

Male C57BL/6 mice (6–8 weeks old) were purchased from Beijing Vital River Laboratory Animal Technology Co., Ltd., China. Mice were housed in a specific-pathogen-free environment maintained at constant temperature and humidity and fed standard laboratory animal chow and water. Animal experiments were approved by the Ethics Committee of Xiamen University Medical School.

### 2.3 Cell culture

H9C2 cells are cultured in Dulbecco’s Modified Eagle Medium with high glucose medium (Gbico, United States), supplemented with 10% fetal bovine serum (Sigma, United States) and 1% streptomycin-penicillin (Gbico, United States), and maintained at 37°C with 5% CO_2_.

### 2.4 Synthesis of poly (DH-Se/PEG/PPG urethane)

Poly (DH-Se/PEG/PPG urethane) (Se-PEG-PPG) was synthesized by a modified method developed by our group (Luo et al., 2019). Briefly, used 0.05 g purified DH-Se compound, 10 g short-chain PEG, and 5 g PPG as starting materials. These raw materials were first vacuum-dried at 40°C, followed by azeotropic distillation with anhydrous toluene to remove trace water. Then, under the protection of argon, they were heated in an oil bath at 75°C, after which 0.913 g 1.6-hexadi-diisocyanate (HDMI) was added as cross-linker for further 48 h reaction, and the crude product precipitated with ether precipitation and dialysis. Finally, purified Se-PEG-PPG was obtained through dialysis using a 10 kDa pouch, which was then lyophilised.

### 2.5 Determination of nuclear magnetic resonance hydrogen spectra

Se-PEG-PPG (5 mg/ml) was dissolved in a 5 mm nuclear magnetic resonance (NMR) tube containing deuterium chloroform (CDCl_3_), for determination of NMR hydrogen spectra on a Bruker AV-400 NMR spectrometer.

### 2.6 Determination of Fourier Transform infrared spectroscopy spectrum

Fourier Transform infrared spectroscopy (FT-IR) spectrum was recorded on BRUKE TENSORII Fourier Transform infrared spectrometer. FT-IR spectrum of solid sample on KBr pellet was recorded on 32 scans signal-averaged with a resolution of 2 cm^−1^ at room temperature.

### 2.7 Determination of rheological parameters of Se-PEG-PPG

Clear urethane was formed by mixing 200 mg or 300 mg Se-PEG-PPG and 10 ml distilled water in a 15 ml centrifuge tube, following which it was left overnight in a refrigerator at 4°C. The storage modulus (G′) and loss modulus (G″) of the prepared hydrogel at different temperatures were measured using a rheometer (angular frequency was fixed at 10 rad/s).

### 2.8 Scanning electron microscope imaging of Se-PEG-PPG

The Se-PEG-PPG hydrogel was fast frozen by liquid nitrogen, and then was freeze-dried in lyophilizer. Finally, using scanning electron microscopy (GeminiSEM 500) to photograph the samples.

### 2.9 H9C2 cell viability *in vitro*

Briefly, H9C2 cells at logarithmic growth stage were collected and added into 96-well plate at the density of 1 × 10^4^cells/well, incubated overnight in an incubator at 37°C containing 5% CO_2_ and 98% humidity. Replaced the medium in the 96-well plate with fresh medium containing Se-PEG-PPG (*n* = 6) with different concentrations (0, 0.01%, 0.05%, 0.1%, 0.5%, 1%, 2%, 3%, 4%). After 24 h continue incubation, the medium was aspirated, with the addition of 100 μl fresh DMEM containing 0.5 mg/ml MTT [3-(4, 5-dimethyl-2-thiazole)-2, 5-diphenyl tetraazolium thiazole blue] to each well, while another 4 h incubation was conducted before the removal of cutural medium and the further addition of 150 μl DMSO. After that, the absorbance value (OD) of each well was measured with a microplate reader (absorption wavelength: 490 nm, the reference wavelength: 570 nm). Cell survival rate was calculated according to the formula:

$$\text{Cell}\;\text{viability}\left(\%\right)=\left[OD_{\left(\text{test}\right)}-{\text{OD}}_{\left(\text{blank}\right)}\right]/\left[{\text{OD}}_{\left(\text{control}\right)}-{\text{OD}}_{\left(\text{blank}\right)}\right]\times 100\%$$

### 2.10 DPPH free radical scavenging assay

The antioxidant activities of Se-PEG-PPG hydrogel was determined *in vitro* using 2,2-diphenyl- 1-Picrylhydrazyl (DPPH). Briefly, 6% Se-PEG-PPG hydrogel was continuously diluted to different concentrations (0.5%, 1.0%, 2%, 3%). DPPH solution was freshly prepared with absolute ethanol (100 µM). Then 100 μl polymer solution was added to DPPH absolute ethanol solution. The mixture was then vortexed and placed in the dark for 30 min at room temperature. DPPH/ddH_2_O was utilized as a positive control group, while DPPH/Vitamin C was used as a positive control group. Then, mixtures were measured at *λ* of 400–800 nm using an ultraviolet-visible spectrophotometer.

### 2.11 Distribution of hydrogel in myocardial tissue

Firstly, Se-PEG-PPG-FITC was synthesized. In details, accurately weigh 20 mg of above synthesized Se-PEG-PPG and 30 mg of FITC, added 20 ml of DMSO to dissolve and adjusted the pH to 8.0 with NaHCO3, then stirred for 48 h at room temperature in the dark. After that, dialyzed with a 5000 MWCO dialysis bag for 48 h and freeze-dried. Secondly, accurately weigh 4 mg of synthesized Se-PEG-PPG-FITC was dissolved with 200 µl distilled water in a 2 ml centrifuge tube, and left overnight out of the light in a refrigerator at 4°C. Then synthesized Se-PEG-PPG-FITC (20 µl) was injected into left ventricle at multiple points with Hamilton injection needle. After 30 min, the ventricles of mice were obtained and fixed in O.C.T, and sectioned at a thickness of approximately 5 μm with a cryostat (Leica Microsystem, Wetzlar, Germany) and thaw-mounted onto a glass slide. Images were acquired using a Zeiss LSM5 confocal microscope.

### 2.12 Preparation of myocardial infarction model *in vivo*

Mice (22–23 g) were anesthetised *via* intraperitoneal injection of 3.3% chloral hydrate (0.1 ml/10 g) and fixed in supine position. The heart was exposed through a left thoracotomy at the fourth intercostal space. A knot was tied around the left anterior descending coronary artery (LAD) under a surgical microscope. These mice experienced LAD ligation were randomly divided into MI, Se-PEG-PPG group and Pluronic F127 (PF127) group. Mice in the Se-PEG-PPG group with a successfully ligated LAD were injected with 20 μl of 2% Se-PEG-PPG hydrogel at multiple points in the myocardial infarction region below the ligation line, using a Hamilton injection needle. Mice in the PF127 group were injected with 20 μl of 2% PF127 and subjected to the same procedures as those in the Se-PEG-PPG group. Sham-operated (sham) animals were subjected to the same surgical procedures except that the knot was not tied.

### 2.13 Echocardiographic analysis

A Vevo 2100 high-resolution microimaging system (Visual sonic, Toronto, Ontario, Canada) was used to evaluate cardiac function. The operation details of the experiment are the same as in our previous study (Verma et al., 2021). Left ventricular ejection fraction (EF), fractional shortening (FS), left ventricular internal dimension-diastole (LVIDd), and left ventricular internal dimension-systole (LVIDs) were obtained. Each measurement was averaged over at least three consecutive cardiac cycles.

### 2.14 Quantitative real-time PCR

The whole experimental process of the real-time PCR was performed as previously described (Verma et al., 2021). Primer sequence used was as follows: GAPDH, 5′-GGC​AAG​TTC​AAT​GGC​ACA​GT-3′ (forward) and 5′-CGGCATCGAAGGTG GAAGAGTG-3′ (reverse); Collagen I, 5′-GAA​ACC​CGA​GGT​ATG​CTT​GA-3′ (forward) and 5′-GGGTCCCT CGACTCCTACAT-3′ (reverse); Collagen III, 5′-AGC​CAC​CTT​GGT​CAG​TCC​TA-3′ (forward) and 5′-GTG​TAG​AAG​GCT​GTG​GGC​AT-3′ (reverse); α-SMA, 5′-ACT​GGG​ACG​ACA​TGG​AAA​AG-3′ (forward) and 5′-CAT​CTC​CAG​AGT​CCA​GCA​CA-3′ (reverse).

### 2.15 Western blotting

The left ventricle under the ligation was used to measure the expression of protein. The whole experimental process of the Western blotting was performed as previously described (Verma et al., 2021). Antibodies used were as following: Tublin (cat. no. 00082687, Proteintech), α-Smooth muscle actin (α-SMA) (cat. no. 14395-I-AP, Proteintech), Collagen Ⅲ (cat.no. 30565S, Cell Signaling Technology), and Collagen Ⅰ (cat.no. NB600-450, Novus). The result was obtained using an ECL detection kit (Millipore, United States).

### 2.16 Histopathological studies

Paraffin was used to embed left and right ventricles at the level of papillary muscle, followed by cutting of the embedded myocardial tissue into sections of 4 μm thickness. Masson trichrome staining kit (cat.no. D026-1-3, Nan Jing Jian Cheng, China) was used to assess the degree of fibrosis. Photomicrographs were taken using the intelligent biological microscope (OLYMPUS.BX53).

### 2.17 ELISA assay

Mouse ELISA kits (eBioscience, San Diego, CA, United States) were used to measure the levels of proinflammatory cytokines, namely, IL-6 (cat.no. 88-7064-86), TNF-α (cat.no. 88-7324-86), and IL-1β (cat.no. 88-7013-86) in the serum. The whole process was carried out in strict accordance with the instructions in the ELISA kit.

### 2.18 Statistical analysis

All results are presented as mean ± standard error of the mean (SEM) using GraphPad (version 8.0.1, GraphPad Prism Software). One-way ANOVA followed by Bonferroni’s post-hoc test was performed to analyse differences with group comparison. Values with *p* < 0.05 were considered statistically significant.

## 3 Results

### 3.1 Synthesis and characterisation of Se-PEG-PPG

**FIGURE 1:**
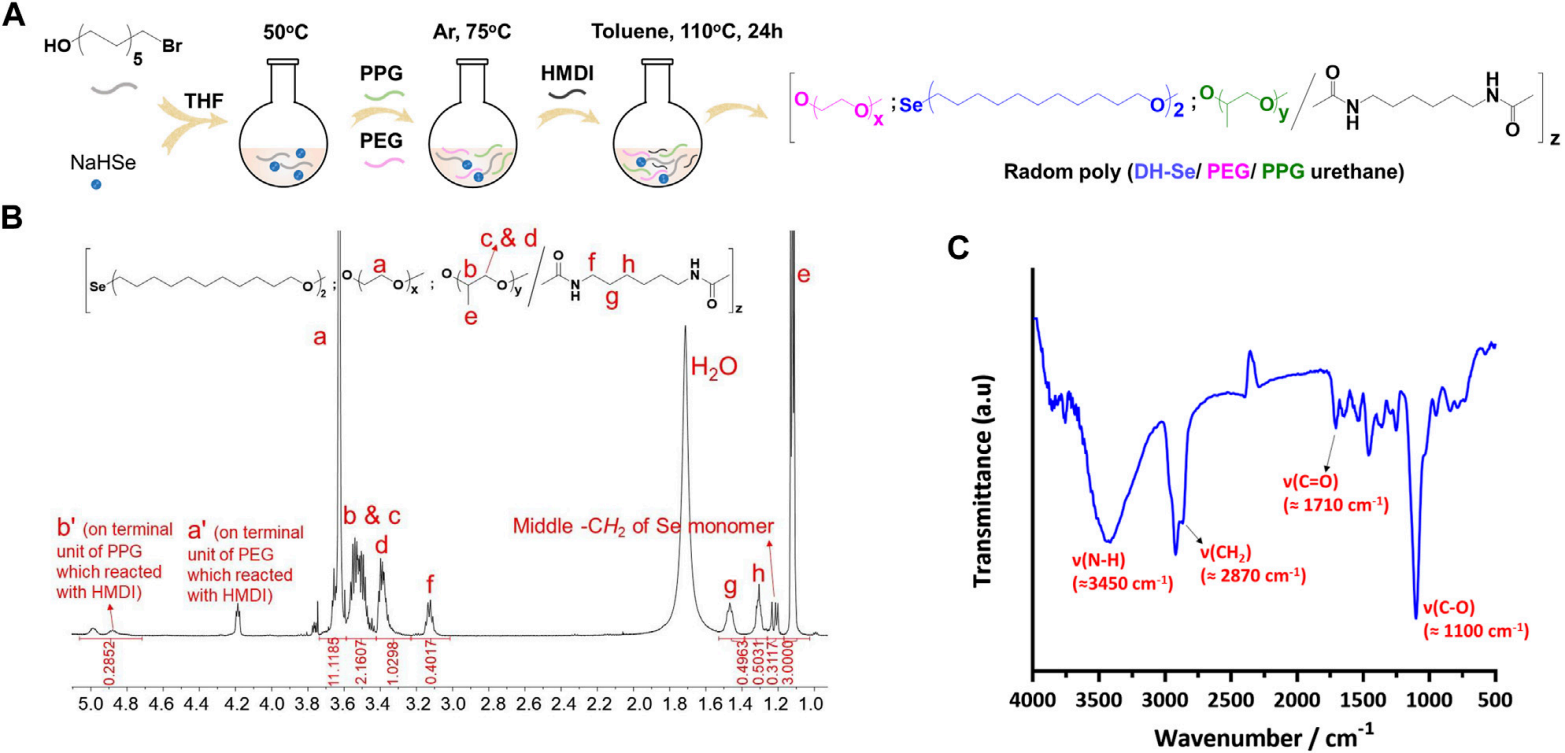
Synthesis and characterization of Se-PEG-PPG. **(A)** Synthesis pathway of Se-PEG-PPG; **(B)** ^1^HNMR spectrum of Se-PEG-PPG with detailed peak distribution; **(C)** FT-IR spectrum of Se-PEG-PPG.

In this study, Se-PEG-PPG is essentially a random copolymer, which formed from blends of thermosensitive PPG, water-soluble macromolecular PEG, and redox active DH-Se with HDMI as the crosslinker. The synthesis process is shown in Figure 1A. ^1^H NMR was used for the detection of any structural defect produced during the synthesis process. As shown in Figure 1B, the proton chemical shift of -CH2- in PEG appeared at *δ* = 3.6 ppm (peak a); the chemical shift at 4.2 ppm (peak a') was from the terminal -CH2, the reactive site in PEG that enabled crosslinking with HDMI. Peaks b–d, at 3.5–3.3 ppm, were from -CH or -CH2 groups in PPG, peak e at 1.1 ppm was from -CH3 in PPG, and peak b' at 4.9 ppm from the terminal proton reacting with HDMI, suggesting the formation of a crosslink between HDMI and the PPG block. The peak at 1.2 ppm was from the -CH2 proton in the selenium-containing monomer, which indicated that selenium was successfully incorporated into the polymer. Meanwhile, from the spectra, we could calculate the final PEG/PPG ratio by typical peaks a and e, labelled in spectra. Peak a refers to C*H*_2_C*H*_2_ of PEG units, while peak e refers to the C*H*_3_ of PPG units. According to their integration values, we calculated that PEG/PPG ratio is 2.78/1 in copolymer Se-PEG-PPG. Meanwhile, according to the integral values of Se units and PPG units, we calculated that the Se/PPG ratio is 1/9 in the copolymer SE-PEG-PPG. Based on these results, we calculated that the ratio of Se/PEG/PPG was 1/25/9 in copolymer SE-PEG-PPG. And FT-IR spectrum was recorded to verify the chemical structure. As shown in Figure 1C, the characteristic signals of -NH, -C*H*_2_, -C=O and -CO- groups in the copolymer were 3,450, 2,870, 1,710, and 1,100 cm^−1^, respectively. The ^1^HNMR and FT-IR results showed that the copolymer Se-PEG-PPG was successfully synthesised. Molecular characterization was further performed using gel permeation chromatography (GPC) (Luo et al., 2019; Xu et al., 2021).

**FIGURE 2:**
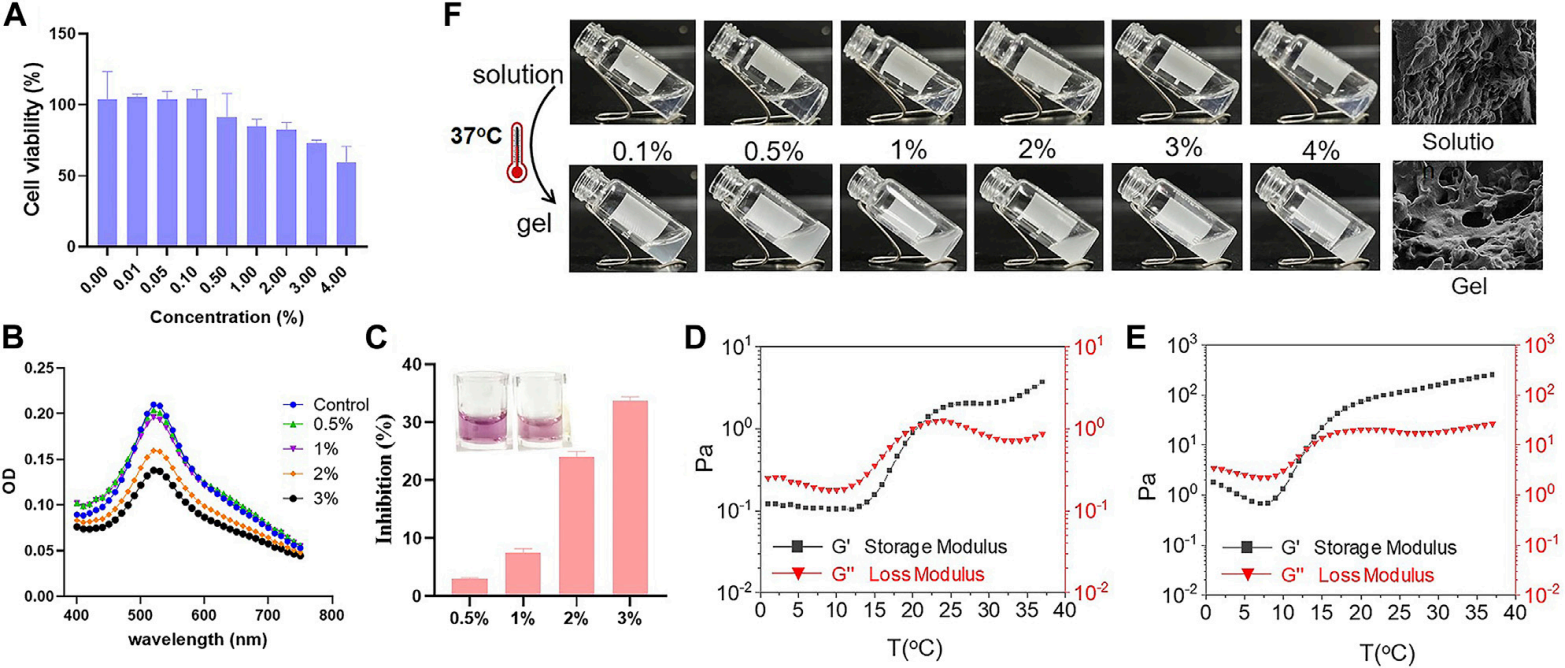
*In vitro* characterization of Se-PEG-PPG. **(A)** H9C2 cell viability after Se-PEG-PPG treatment; **(B)** Antioxidant activity of Se-PEG-PPG hydrogel assessed using DPPH; **(C)** The free radical scavenging rate of different concentrations Se-PEG-PPG measured at *λ* max of 520 nm using DPPH; **(D)** Rheological properties of 2% Se-PEG-PPG; **(E)** Rheological properties of 3% Se-PEG-PPG. Black and red represent storage modulus (G′) and loss modulus (G″), respectively; **(F)** *In vitro* gelation of Se-PEG-PPG hydrogel at 37°C and SEM images of Se-PEG-PPG hydrogel before and after gelation. Scale bar, 10 μm.

Subsequently, cell viability test *in vitro* was conducted to evaluate the safety of Se-PEG-PPG hydrogel. The experimental result showed that, compared with the control group, the activity of cells was no significant difference, indicating that Se-PEG-PPG hydrogel is not significantly toxic to H9C2 cells (Figure 2A). At the same time, because selenium has antioxidant properties, we speculated that Se-PEG-PPG hydrogel might have antioxidant properties. Therefore, DPPH free radical scavenging assay was used to detect antioxidant activity of Se-PEG-PPG hydrogel *in vitro*. The experimental results showed that 2% and 3% hydrogels had obvious clearance effect of DPPH, and the effect was in a dose-dependent manner, indicating that Se-PEG-PPG hydrogel has antioxidant activity (Figure 2B). As 2% and 3% Se-PEG-PPG hydrogels had better free radical scavenging ability (the free radical scavenging rates were 24% and 34%, respectively, Figure 2C).

**FIGURE 3:**
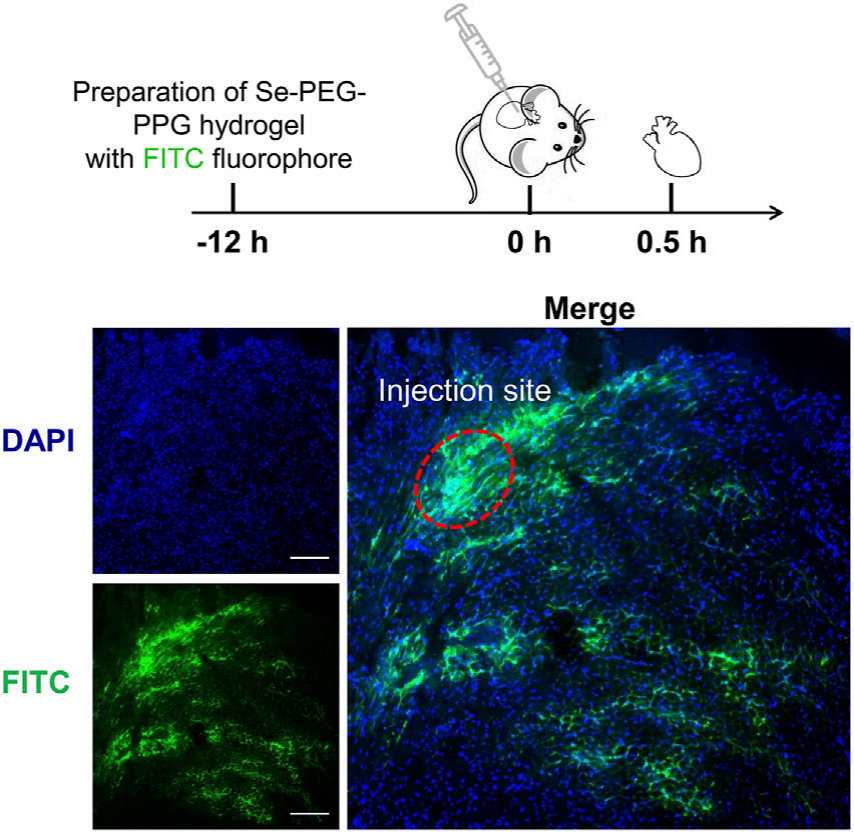
Representative sectioning and imaging of myocardium after hydrogel injection. Blue (DAPI), cell nucleus; green (FITC), Se-PEG-PPG hydrogel; Scale bar, 100 μm.

Then, the rheological properties of 2% and 3% Se-PEG-PPG hydrogel were observed by *in vitro* rheological test. As shown in Figures 2D,E, the gelatinization temperature of 2% and 3% Se-PEG-PPG hydrogel was 21°C and 14°C, respectively. When reaching the gelatinization temperature, Se-PEG-PPG hydrogel changed from solution to hydrogel (Figure 2F). Meanwhile, the ultrastructure of 2% Se-PEG-PPG before and after gelation was observed by SEM, as displayed in Figure 2B. This indicated that Se-PEG-PPG hydrogel had certain mechanical properties *in vivo*, which may provide the MI-affected ventricular wall with additional support. Moreover, it was observed that at 37°C, compared with 236 Pa of 3%, 2% Se-PEG-PPG hydrogel exhibited a low mechanical strength and a storage modulus of 3.71 Pa, which greatly reduced the possibility of secondary myocardial damage. Otherwise, the 2% Se-PEG-PPG hydrogel has a better fluidity, which enables distribution of the gel within the myocardium and solidification *in vivo* directly at the site of interest. And our sectioning and imaging of the tissue data also supported this point (Shown in Figure 3). In short, considering the comprehensive safety and experimental feasibility, 2% Se-PEG-PPG hydrogel was chosen for the following experiments.

### 3.2 Cardiac function recovery through intramyocardial injection with Se-PEG-PPG post MI

**FIGURE 4:**
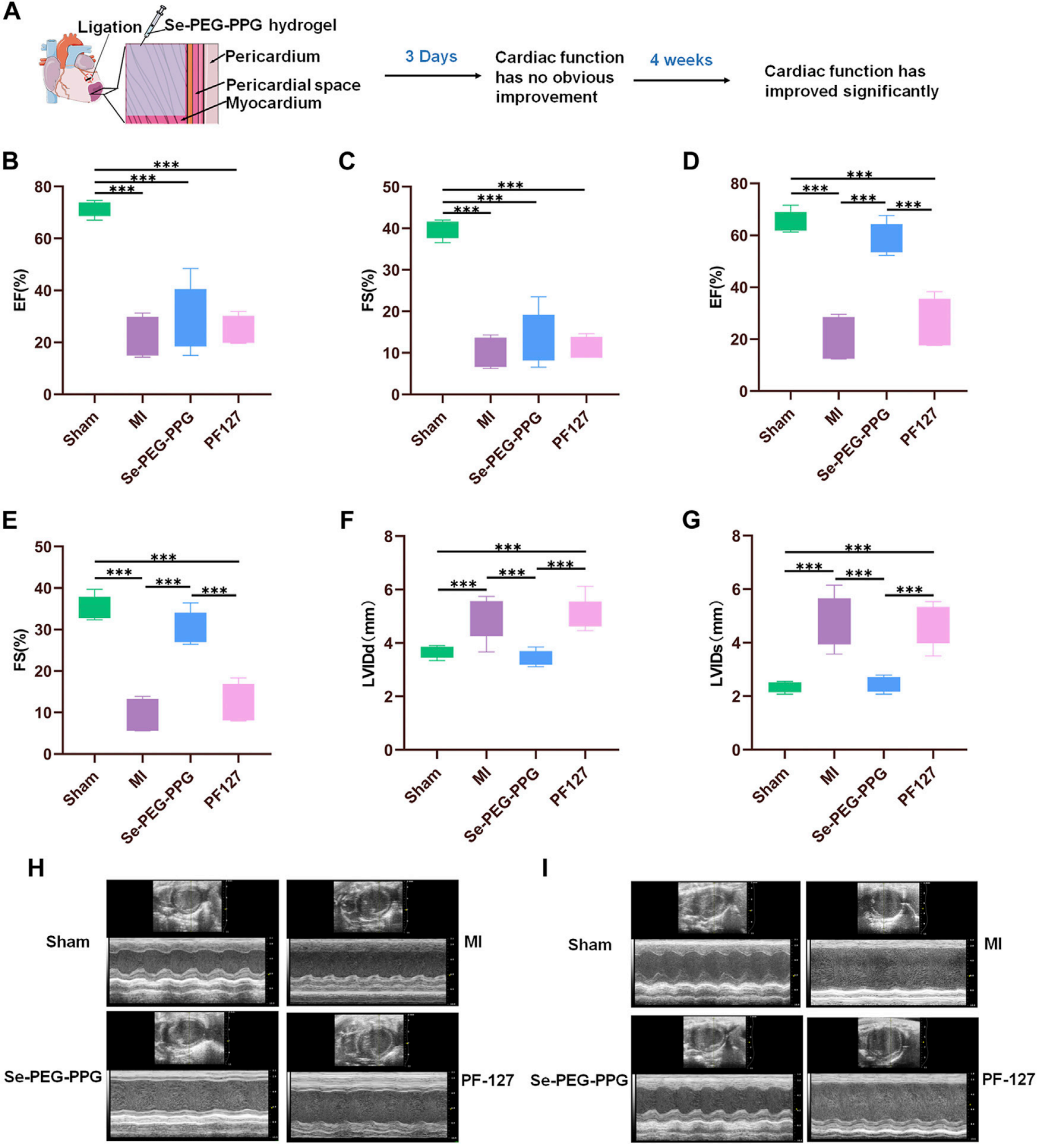
The performance of cardiac function post MI. **(A)** Myocardial function has improved significantly at 28th day post MI; **(B,C)** Quantitative analysis of left ventricular ejection fraction (EF) and shortened fraction (FS) on the third day after MI (*n* = 5, ***p < 0.01); **(D,E)** Quantitative analysis of left ventricular ejection fraction (EF) and shortened fraction (FS) at 28th day post MI (*n* = 5, ***p < 0.01); **(F,G)** Quantitative analysis of left ventricular diastolic inner diameter (LVIDd) and left ventricular systolic inner diameter (LVIDS) at 28th day post MI (*n* = 5, ***p < 0.01); **(H)** Representative echocardiographic images on the third day after MI; **(I)** Representative echocardiographic images at 28th day post MI.

Large-scale tissue loss following MI can induce exacerbation in cardiac function (Song et al., 2016). Recovery of cardiac function is associated with better long-term outcomes after MI (Wu et al., 2020). In this study, recovery of cardiac function was assessed *via* echocardiography (ECG) at day 3 (early phase) and week 4 (later phase) after LAD ligation in the mouse model (Figure 4A). At the same time, in order to better show that our hydrogel has good therapeutic effect, we chose PF127, a commercially available and low toxic hydrogel without physiological activity, as the control. The echocardiographic results at day 3 showed that the percentages of EF and FS were smaller in the MI group when compared with the Sham group (Figures 4B,C), which indicated that the MI mouse model was successfully established. In addition, because the values of EF and FS did not significantly differ among the MI, Se-PEG-PPG, and PF127 groups, it was inferred that Se-PEG-PPG hydrogel did not come into play during the first 3 days after LAD ligation.

After 4 weeks, the MI and PF127 groups showed no significant differences in EF and FS, whereas the Se-PEG-PPG group experienced a marked increase in these values, demonstrating an excellent recovery in the blood-pumping ability of the heart after injection with Se-PEG-PPG hydrogel (Figures 4D,E). Moreover, the “M”-shaped ECG indicated a sharp decrease in LVIDd and LVIDs in the Se-PEG-PPG group when compared with the MI group, suggesting an impressive improvement in ventricular filling (Figures 4F–I). To sum up, the echocardiographic results clearly demonstrated that Se-PEG-PPG hydrogel treatment following MI could effectively promote post-MI recovery of cardiac function.

### 3.3 Significant downregulation of IL-6 expression through intramyocardial injection with Se-PEG-PPG hydrogel post MI

**FIGURE 5:**
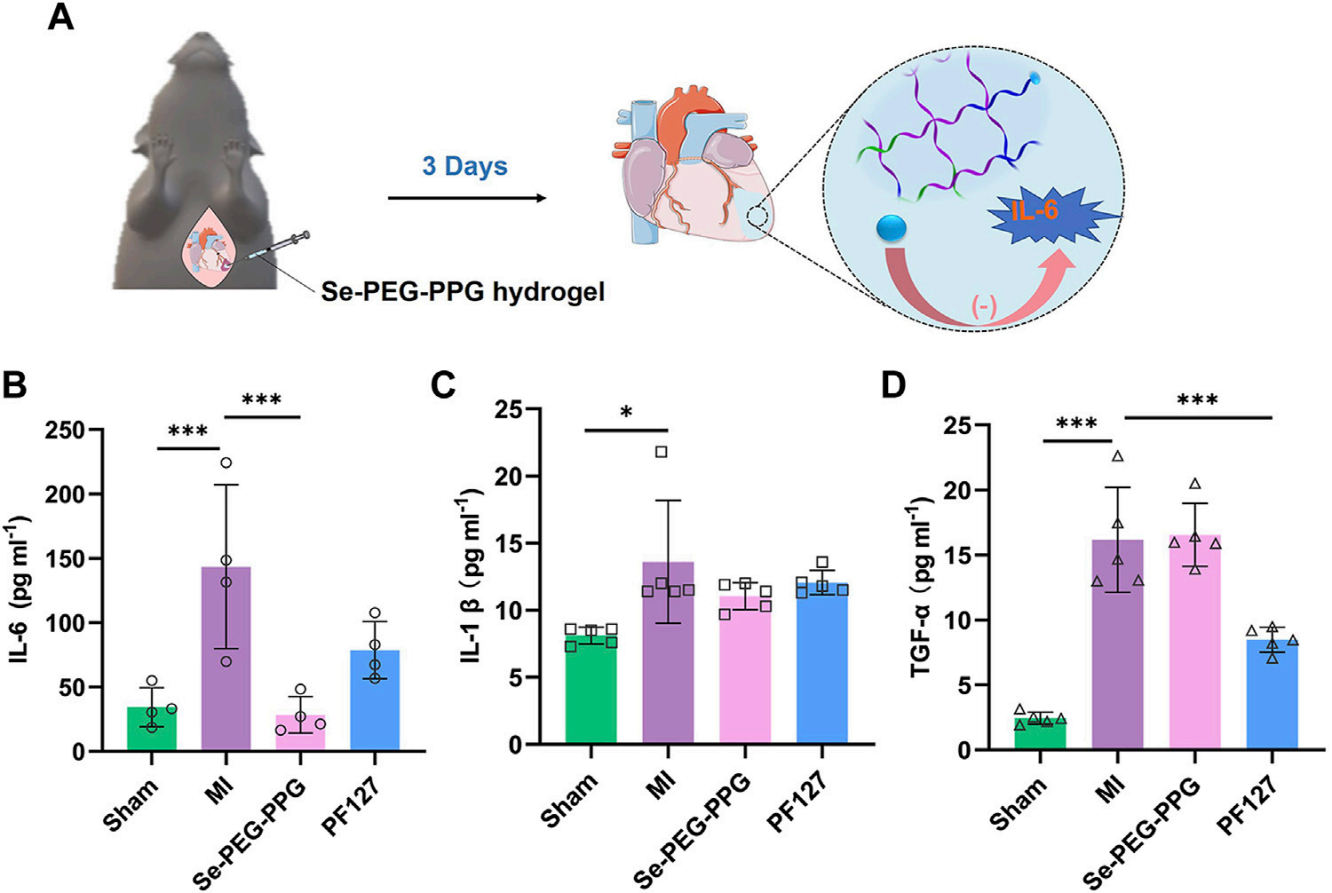
Expression of inflammatory cytokines in serum on the third day after MI. **(A)** Diagrammatic sketch of downregulation of IL-6 expression after intramyocardial injection with Se-PEG-PPG hydrogel; **(B)** Expression level of IL-6 (*n* = 4, ***p < 0.01); **(C)** Expression level of IL-1*β* (*n* = 5, ***p < 0.01); **(D)** Expression level of TNF-α (*n* = 5, ***p < 0.01).

Post-MI hyperinflammatory response is reported to hinder the recovery from MI and to cause adverse remodelling (Song et al., 2016). To study the potential effects of Se-PEG-PPG hydrogel on post-MI inflammatory response, the expression levels of proinflammatory cytokines including TNF-α, IL-6, and IL-1β in serum were measured at day 3 after LAD ligation. Compared with the Sham group, the levels of TNF-α, IL-6, and IL-1β in serum were significantly elevated in the MI group because of massive leukocytic infiltration resulting from myocardial damage. Interestingly, compared with the MI group, IL-6 expression experienced a selective downregulation in the Se-PEG-PPG group (Figures 5A,B), in addition to insignificant changes in the expression levels of TNF-α and IL-1β (Figures 5C,D). Collectively, these results suggested that Se-PEG-PPG hydrogel could induce selective inhibition of IL-6 expression and might be able to suppress inflammation. Considering prior evidence suggesting the significant association of post-MI inflammatory response with the serum level of selenium (Dallak, 2017), the current study presumed that Se-PEG-PPG hydrogel might inhibit IL-6 expression by regulating the level of selenium.

### 3.4 Significant remission of post-MI fibrosis and improvement in left ventricular remodelling through intramyocardial injection with Se-PEG-PPG hydrogel

**FIGURE 6:**
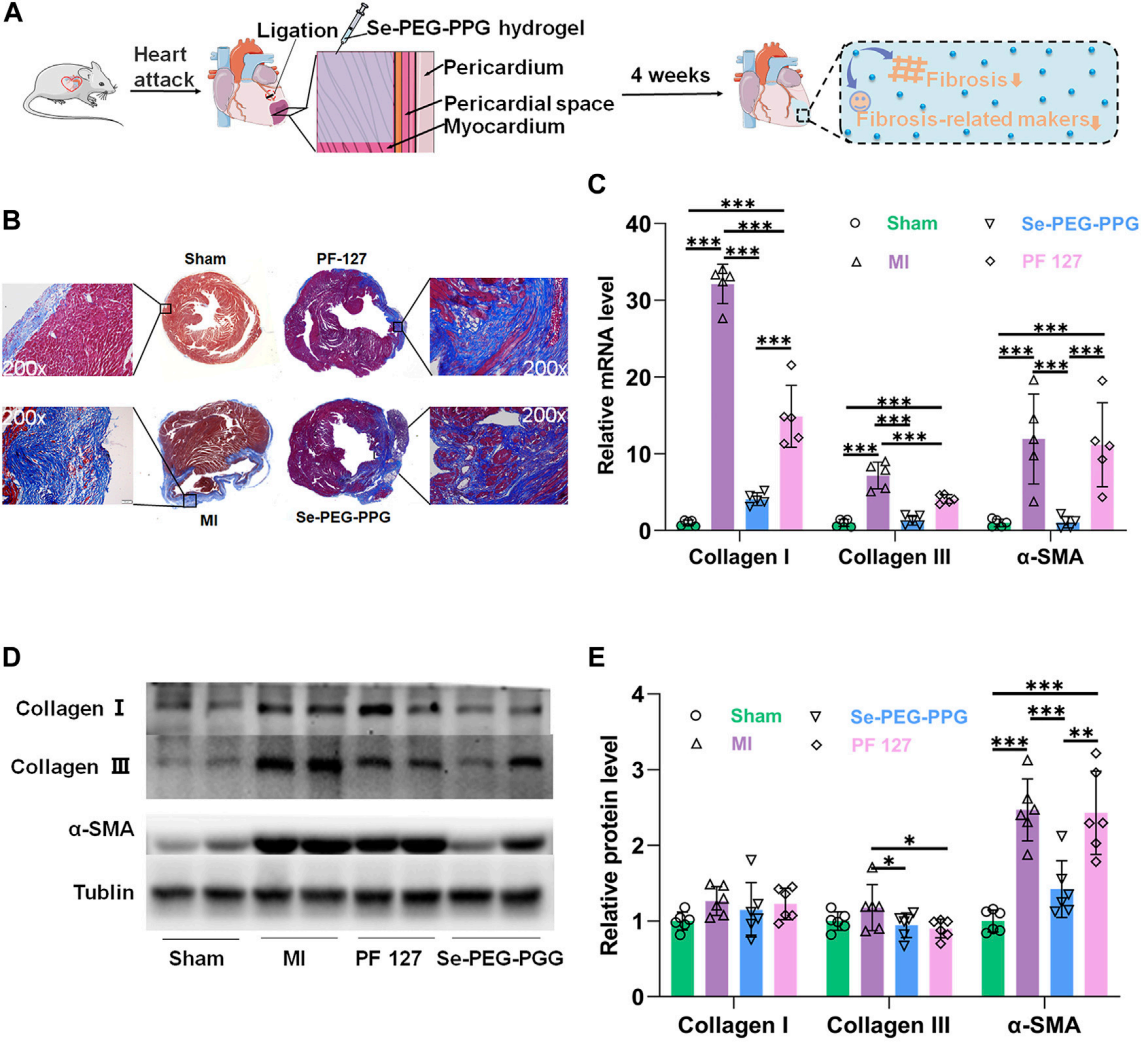
Poly (DH-Se/PEG/PPG urethane) attenuates cardiac fibrosis MI induced. **(A)** Schematic illustration of decreased fibrosis and fibrosis-related makers after injection of Se-PEG-PPG hydrogel; **(B)** Masson’s trichrome staining of heart was shown. Collagen fibers were stained blue and myocardium was stained red. Scale bar: 10 mm; **(C)** Relative mRNA expression levels of α-SMA, Collagen Ⅰ and Collagen Ⅲ were estimated by RT-PCR and normalized to those of GAPDH (*n* = 5, ***p < 0.01); **(D,E)** The protein expression level of α-SMA, Collagen Ⅰ and Collagen Ⅲ in mouse cardiac tissues (*n* = 6, ***p < 0.01).

Cardiac myocytes undergo irreversible death when MI occurs, leading to the formation of non-contractile scars, collagen deposition, and ECM degradation, eventually leading to non-adaptive changes in the shape, structure, and function of the heart, which is termed “left ventricular remodelling” (van den Borne et al., 2010). The left ventricular structure and physiological condition, as well as long-term survival of the patient, are directly associated with the degree of post-MI left ventricular remodelling (Ferrante and Stefanini, 2017). Therefore, Masson staining and the gene and protein expression levels of fibrosis related proteins were performed to determine whether Se-PEG-PPG hydrogel could relieve left ventricular remodelling in mice in the MI group (Figure 6A). As shown in Figure 6B, the Masson staining results at week four revealed that compared with the Sham group, the left ventricle of the MI group was largely occupied by collagen tissue, and the left ventricular wall had become much thinner. Compared with the MI group, the Se-PEG-PPG group had a significantly thicker left ventricular wall and less collagen tissue in the infarct area. The Masson staining results suggested that intramyocardial injection (IMI) with Se-PEG-PPG hydrogel provided effective structural base support for the post-MI heart. This protective effect is probably associated with the significant inhibition of IL-6 expression by Se-PEG-PPG hydrogel; as a typical proinflammatory cytokine, IL-6 is highly active in triggering inflammation (Shahrivari et al., 2017), and thus, the inhibition of IL-6 can prevent adverse ventricular remodelling after MI (Jing et al., 2019).

Moreover, the gene and protein expression levels of collagen I, collagen III, and α-SMA, at week four after LAD ligation, were determined to determine the potential role of Se-PEG-PPG hydrogel in the process of post-MI fibrosis. The gene expression analysis suggested that the MI group saw a marked upregulation of collagen I, collagen III, and α-SMA mRNA expression, which was significantly inhibited following the use of Se-PEG-PPG hydrogel (Figure 6C); this demonstrated the inhibitory effects of Se-PEG-PPG hydrogel on the expression of fibrosis-associated genes. As for protein expression, compared with the MI group, the Se-PEG-PPG group exhibited selective downregulation of protein expression in α-SMA and collagen III (Figures 6D,E). As for the different changes of genes and proteins in hydrogels, we speculated it may be due to the existence of post-transcriptional control. Overall, relevant data suggested that IMI with Se-PEG-PPG hydrogel could substantially reduce the risk of post-MI myocardial fibrosis.

## 4 Discussion

Various cardiovascular diseases, including MI, are still among the primary causes of human death. Among various therapeutic methods for the treatment of MI, traditional drug treatment is the basic treatment, but the type and number of drugs are less at present. In order to find more therapeutic methods to treat myocardial infarction, injectable hydrogels appeared in our minds. Injectable hydrogels have attracted the attention of many researchers, owing to having advantages such as mechanical support for the injured myocardium, ability to deliver protective factors and drugs, and minimally invasive injection (Ding et al., 2020).

In this study, the main chain of the novel selenium-containing polymer hydrogel—Se-PEG-PPG, which was synthesised using DH-Se, PPG, and PEG fragments and applied to a mouse MI model *in vivo*—would undergo solution-to-gelation (sol-to-gel) transition due to the increase in temperature. A copolymer, which has a sol-to-gel transition induced by thermal stimulation, has unique advantages, because it will not cause damage to cardiomyocytes and not need to be activated *via* photoinduction (e.g., using UV photoinduction) (Noshadi et al., 2017). Thus, in general, the potential harm from the polymer is less. In addition, the Se-PEG-PPG hydrogel used in this study has certain mechanical properties (Figure 2D). Therefore, it can adapt to the irregularity of the damaged site by *in-situ* gelation a for the injured myocardium to compensate for tissue damage.

An investigation on the effects of Se-PEG-PPG hydrogel at different injection concentrations on the recovery of cardiac function in mice in the MI group (MI mice) (including at 0.5%, 1%, 2%, and 3%, as seen in Supplementary Figure S1) was studied. It indicated that 0.5% Se-PEG-PPG hydrogel resulted in no significant improvement in cardiac function (Supplementary Figure S1), probably because 0.5% Se-PEG-PPG hydrogel contained less selenium, thereby reducing the therapeutic effect. However, 1% and 2% Se-PEG-PPG hydrogel presented significant improvements in cardiac function in MI mice, mainly showing that the values of EF and FS increased significantly (Supplementary Figure S1). It was also established that it was difficult to administer an IMI using 3% Se-PEG-PPG hydrogel because of its high viscosity. Therefore, considering the ease of administering the IMI and the improvement of cardiac function, 2% Se-PEG-PPG hydrogel, which is relatively easy to inject and can significantly improve the cardiac function of MI mice, was finally chosen as the concentration for the formal experiment.

Selenium is an important component of Se-PEG-PPG hydrogel used in this study. Previously, researchers have found that exogenous selenium can significantly resolved cardiac systolic dysfunction in various animal models of cardiovascular diseases (Venardos et al., 2004; Bordoni et al., 2005; Ostadalova et al., 2007). Its deficiency will increase the expression of fibrotic markers (such as type I and type III collagen); the biomarkers of myocardial fibrosis in patients with MI will also be reduced after selenium supplementation (Alehagen et al., 2018; Lin et al., 2022). Moreover, selenium can inhibit the production of interleukins and TNF-α by regulating inflammatory signalling pathways (Dallak, 2017). Therefore, we analysed the cardiac function, ventricular remodelling, fibrosis, and inflammatory factors in MI mice after Se-PEG-PPG hydrogel injection, revealing that 4 weeks after Se-PEG-PPG hydrogel treatment, cardiac function was significantly restored, ventricular remodelling was significantly improved, and fibrosis of infarction region and the serum expression of IL-6 were effectively inhibited in MI mice. The aforementioned results clearly demonstrate that IMI with Se-PEG-PPG hydrogel can play an anti-inflammatory role in acute stages of MI and significantly improve the cardiac function in mice with late-stage MI.

Because the muscle of heart is a complex, conductive, and contracting muscle, the ideal material used for treatment should possess biocompatibility, conductivity, and mechanical strength similar to the heart, as well as resist the harsh conditions caused during myocardial injury (Peña et al., 2018). The Se-PEG-PPG hydrogel used in the current study has some shortcomings despite its benefits. For example, it is not firm enough to provide continuous mechanical support for the damaged heart tissue. In future, the mechanical properties of the materials we use can be further strengthened by changing the amount of polymer dissolved in aqueous solution or by using monomers at different concentrations to change the chemical composition of the polymer itself. Overall, the hydrogel prepared in this study has the following advantages: minimal damage caused to cardiomyocytes, adaptability to the irregularity of the injury site, and no limitation on activity when there is massive loss of transplanted cells. It has been applied for cardiac repair in mice with MI and exhibited good therapeutic effects.

## 5 Conclusion

In this study, Se-PEG-PPG, a novel selenium-containing polymeric hydrogel demonstrated to have therapeutic effects on mice afflicted with MI, was formulated. It was discovered that IMI with Se-PEG-PPG hydrogel could dramatically improve post-MI cardiac function. Moreover, this study presents a preliminary analysis of the role of Se-PEG-PPG hydrogel in inflammation and fibrosis and suggests that IMI with Se-PEG-PPG hydrogel could reduce the secretion of proinflammatory cytokines, ameliorate myocardial fibrosis, and improve left ventricular remodelling. This contributed to our understanding of the mechanism responsible for the supportive properties of Se-PEG-PPG hydrogel in the recovery of myocardial function. In conclusion, Se-PEG-PPG is a novel and innovative biomaterial that helps promote the recovery of post-MI cardiac structure and function, as well as offers a new strategy and basis for potentially treating MI and other ischemic diseases. This approach also presents a potential adjuvant therapy for coronary artery stent placement or bypass grafting.

## Data Availability

The original contributions presented in the study are included in the article/Supplementary Material, further inquiries can be directed to the corresponding authors.

## Abbreviations

DH-Se, di-(1-hydroxylyndecyl) selenide; ECG, echocardiography; ECM, extracellular matrix; EF, left ventricular ejection fraction; ELISA, enzyme-linked immunosorbent assay; EPO, erythropoietin; FS, fractional shortening; FT-IR, fourier transform infrared spectroscopy; HDMI, 1.6-hexadi-diisocyanate; hiPSC-CMs, cardiomyocytes derived from human induced pluripotent stem cell; IL-1*β*, interleukin-1*β*; IL-6, interleukin-6; IMI, intramyocardial injection; LAD, left anterior descending coronary artery; LVIDd, left ventricular internal dimension-diastole; LVIDs, left ventricular internal dimension-systole; MI, myocardial infarction; NMR, nuclear magnetic resonance; PCR, polymerase chain reaction; PEG, polypropylene glycol; PF127, pluronic F127; PPG, polyethylene glycol; Se, selenium; SEM, scanning electron microscope; sol-to-gel, solution-to-gelation; TNF-α, tumor necrosis factor-α.
