# Supplementary material for: Injectable selenium-containing polymeric hydrogel formulation for effective treatment of myocardial infarction
Source: Front Bioeng Biotechnol. 2022 Jul 11;10:912562. doi: 10.3389/fbioe.2022.912562 (PMC9403312; doi:10.3389/fbioe.2022.912562)
Supplement: Supplementary file 1 [file DataSheet1.DOCX]

**Supporting Information**

**Injectable selenium-containing polymeric hydrogel formulation for effective treatment of myocardial infarction**

Cui yang^a, †^, Chunyan zhu^b, †^, Yanling Li^a^, Zhenghao Zhang^a^, Jiajia Xu^a^, Minwei Chen^a^, Runjing Li^a^, Shixiao Liu^a^, Yunlong Wu^b*^, Zhengrong Huang^a*^, Caisheng Wu^b*^

^a^Department of Cardiology, Xiamen Key Laboratory of Cardiac Electrophysiology, Xiamen Institute of Cardiovascular Diseases, The First Affiliated Hospital of Xiamen University, School of Medicine, Xiamen University, Xiamen 361003, China

^b^Fujian Provincial Key Laboratory of Innovative Drug Target Research and State Key Laboratory of Cellular Stress Biology, School of Pharmaceutical Sciences, Xiamen University, Xiamen 361102, China

***Corresponding author:**

**Caisheng Wu**: [wucsh@xmu.edu.cn](mailto:wucsh@xmu.edu.cn);

**Zhengrong Huang:** [huangzhengrong@xmu.edu.cn](mailto:huangzhengrong@xmu.edu.cn)**;**

**Yunlong Wu:** [wuyl@xmu.edu.cn](mailto:wuyl@xmu.edu.cn)**;**

^†^These authors have contributed equally to this work and share first authorship

Table S1. Molecular characteristics and thermal properties of poly(DHSe/PEG/PPG urethane).

| Sample | Feed Ratio | | Thermal Analysis | | | GPC | |
| --- | --- | --- | --- | --- | --- | --- | --- |
|  | PEG | PPG | *T*_m_ ^a^/ ^O^C | *T*_g_ ^a^ / ^O^C | *T*_d_ ^b^ / ^O^C | M_n_/Da | *Ð* |
| DHSe-1 1 | 1 | 1 | 41.85 | -57.11 | 298 | 32,018 | 1.14 |
| DHSe-3 1 | 3 | 1 | 43.26 | -55.77 | 332 | 25,523 | 1.09 |

**
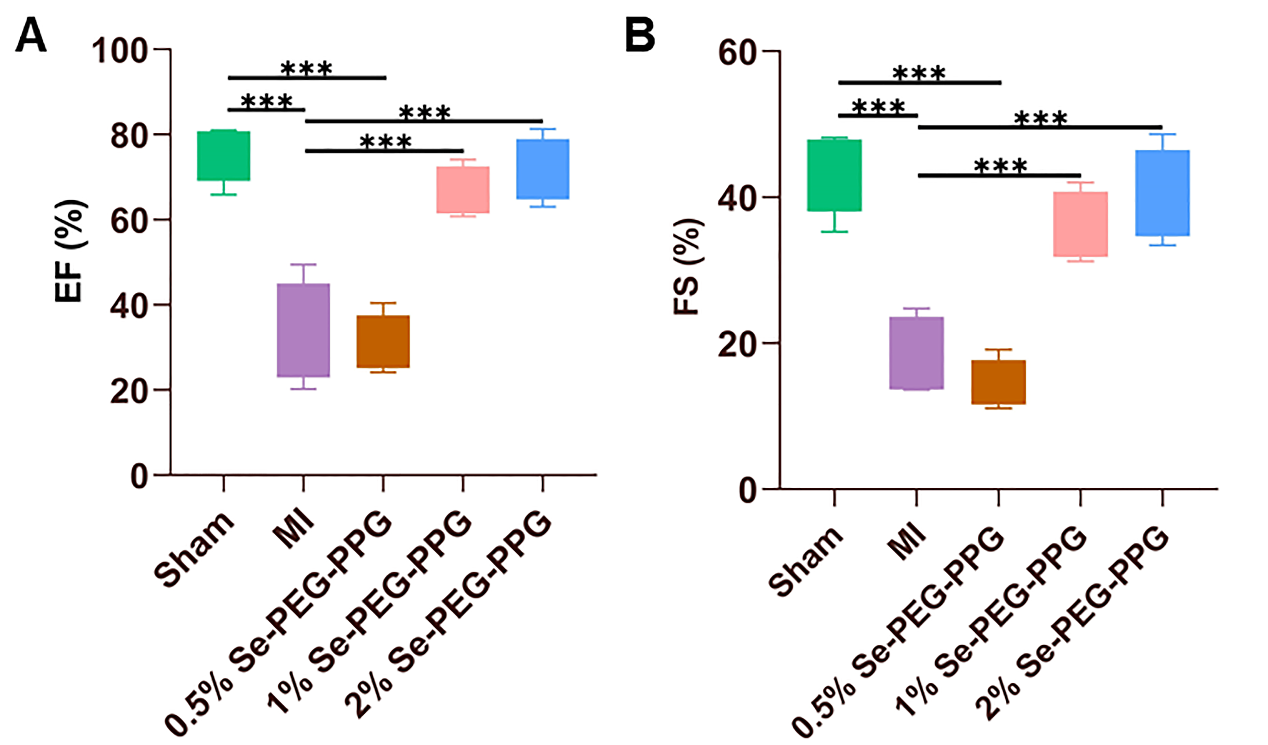
**

**Figure. S1.** Different concentrations of poly (DH‐Se/PEG/PPG urethane) effects on myocardial function after MI. A) and B) Quantitative analysis of left ventricular ejection fraction (EF) and shortened fraction (FS) (n=4, ****P*<0.01). 0.5% Se-PEG-PPG, 1% Se-PEG-PPG and 2% S e-PEG-PPG represent poly (DH‐Se/PEG/PPG urethane) with concentration of 0.5%, 1% and 2%, respectively.


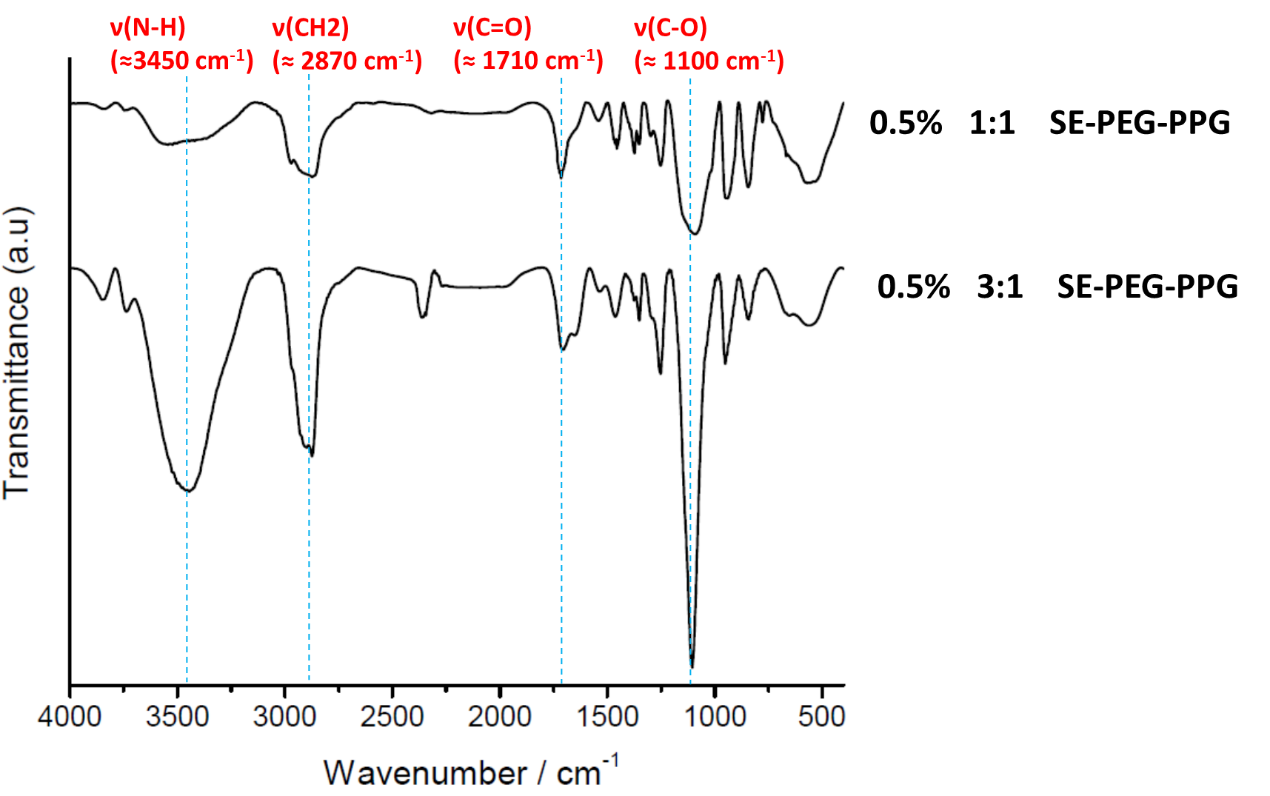


Figure S2. FT-IR curves of poly(DH-Se/PEG/PPG urethane).
